# Assessing preoperative risk of STR in skull meningiomas using MR radiomics and machine learning

**DOI:** 10.1038/s41598-022-18458-4

**Published:** 2022-08-18

**Authors:** Manfred Musigmann, Burak Han Akkurt, Hermann Krähling, Benjamin Brokinkel, Dylan J. H. A. Henssen, Thomas Sartoretti, Nabila Gala Nacul, Walter Stummer, Walter Heindel, Manoj Mannil

**Affiliations:** 1 University Clinic for Radiology, Westfälische Wilhelms-University Muenster and University Hospital Muenster, Albert-Schweitzer-Campus 1, E48149 Muenster, Germany; 2 Department of Neurosurgery, Westfälische Wilhelms-University Muenster and University Hospital Muenster, Albert-Schweitzer-Campus 1, E48149 Muenster, Germany; 3 Department of Medical Imaging, Radboud University Medical Center, Radboud University, 6500HB Nijmegen, The Netherlands; 4 Faculty of Medicine, University of Zürich, Zürich, Switzerland; 5 The Institute of Diagnostic and Interventional Radiology, University Hospital Zurich, University of Zurich, Zurich, Switzerland; 6 Department of Radiology and Nuclear Medicine, Maastricht University Medical Center, Maastricht University, Maastricht, The Netherlands

**Keywords:** Cancer, Surgical oncology

## Abstract

Our aim is to predict possible gross total and subtotal resections of skull meningiomas from pre-treatment T1 post contrast MR-images using radiomics and machine learning in a representative patient cohort. We analyse the accuracy of our model predictions depending on the tumor location within the skull and the postoperative tumor volume. In this retrospective, IRB-approved study, image segmentation of the contrast enhancing parts of the tumor was semi-automatically performed using the 3D Slicer open-source software platform. Imaging data were split into training data and independent test data at random. We extracted a total of 107 radiomic features by hand-delineated regions of interest on T1 post contrast MR images. Feature preselection and model construction were performed with eight different machine learning algorithms. Each model was estimated 100 times on new training data and then tested on a previously unknown, independent test data set to avoid possible overfitting. Our cohort included 138 patients. A gross total resection of the meningioma was performed in 107 cases and a subtotal resection in the remaining 31 cases. Using the training data, the mean area under the curve (AUC), mean accuracy, mean kappa, mean sensitivity and mean specificity were 0.901, 0.875, 0.629, 0.675 and 0.933 respectively. We obtained very similar results with the independent test data: mean AUC = 0.900, mean accuracy = 0.881, mean kappa = 0.644, mean sensitivity = 0.692 and mean specificity = 0.936. Thus, our model exposes good and stable predictive performance with both training and test data. Our radiomics approach shows that with machine learning algorithms and comparatively few explanatory factors such as the location of the tumor within the skull as well as its shape, it is possible to make accurate predictions about whether a meningioma can be completely resected by surgery. Complete resections and resections with larger postoperative tumor volumes can be predicted with very high accuracy. However, cases with very small postoperative tumor volumes are comparatively difficult to predict correctly.

## Introduction

Meningiomas are mostly benign, extra-axial tumors originating from the arachnoid cap cells. They represent 13–26% of all intracranial tumors^1^. The annual incidence of meningiomas in the United States is 5.3 per 100,000 people and increases steadily with age^2^.

According to international guidelines and current literature, primary therapy of meningiomas consists of surgery and adjuvant radiotherapy^3^. Further treatment modalities include systemic and targeted therapies. Following the National Comprehensive Cancer Network (NCCN) guidelines for meningiomas (http://nccn.org/), chemotherapy is only recommended for recurrent (progressive) disease when radiation therapy or further surgical resection is not feasible^4^.

An important aspect impacting the further prognosis and therapy planning concerns the extent of resection (EOR) during initial surgical treatment of the tumor. Specifically, subtotal resection is often associated with recurrent tumor growth and thus with the risk of disease progression^5^. For further therapy planning, it is therefore important to determine as early as possible whether a tumor can be completely resected or not.

Recent studies suggest that machine learning algorithms can be very helpful in answering such clinical questions. For example, machine learning algorithms can improve long-term outcome prediction for patients with ischemic stroke^6^ or predict malaria disease based on patient information^7^. Machine learning algorithms can also be used for a preoperative, non-invasive determination of meningioma grade^8^.

Given the considerations outlined above, the aim of our study is to predict possible gross total resections (GTR) of meningiomas based on pre-treatment MR images and machine learning backed radiomics, and to distinguish these cases from those in which only subtotal resection (STR) can be performed.

## Materials and methods

This study was performed in compliance with the Declaration of Helsinki and approved by the local ethics committee (Ethikkommission der Ärztekammer Westfalen Lippe and University of Muenster, Muenster, Germany). Due to the retrospective nature of the study, written informed consent was waived by Ethikkommission der Ärztekammer Westfalen Lippe and University of Muenster, Muenster, Germany.

Our aim was to distinguish meningioma cases in which a gross total resection of the tumor was possible from those in which only a subtotal resection could be performed. Therefore, we retrospectively searched our database for patients diagnosed with meningioma followed by resection between February 2015 and July 2018. 167 patients were initially screened of which 138 (mean age of 58.86 years) were finally included in our analyses. We excluded 29 patients with (1) missing or non-diagnostic pre-treatment cerebral magnetic resonance imaging, (2) insufficient diagnostic imaging quality, (3) incomplete clinical data, (4) inconsistent histopathology and (5) insufficient follow-up examinations. Most of these 29 patients excluded had incomplete or inconsistent clinical data.

Our final cohort of 138 patients comprises 100 females and 38 males. A gross total meningioma resection (GTR) was achieved in 107 cases. The remaining 31 of the 138 patients had a subtotal resection (STR). We classify GTR cases as those with a postoperative tumor volume (POTV) = 0 cm^3^ and correspondingly STR cases with POTV > 0 cm^3^. Usually, GTR and STR cases are distinguished using the Simpson grade. GTR cases have the Simpson grades I, II and III and STR cases have grades IV and V. In addition to this purely binary distinction between GTR cases (POTV = 0 cm^3^) and STR cases (POTV > 0 cm^3^), we also examine the 138 cases as a function of their exact POTV.

### Image data

We searched the Picture Archiving and Communication System (PACS) of our hospital for cases of meningiomas between February 2015 and July 2018. As a tertiary referral centre, around 33% of patients had external MR imaging. The images were obtained on common 1.5 and 3 T MR scanners of the vendors Philipps Healthcare, Siemens Healthineers and GE Healthcare. We downloaded 3D T1 post Gadolinium images in DICOM format and pseudonymized the DICOM header.

### Radiomics

For purpose of pre-processing, the following parameters were selected: normalize: true, normalizeScale: 100, resampledPixelSpacing: [2, 2, 2], binWidth: 5, voxelArrayShift: 300.

**Figure 1:**
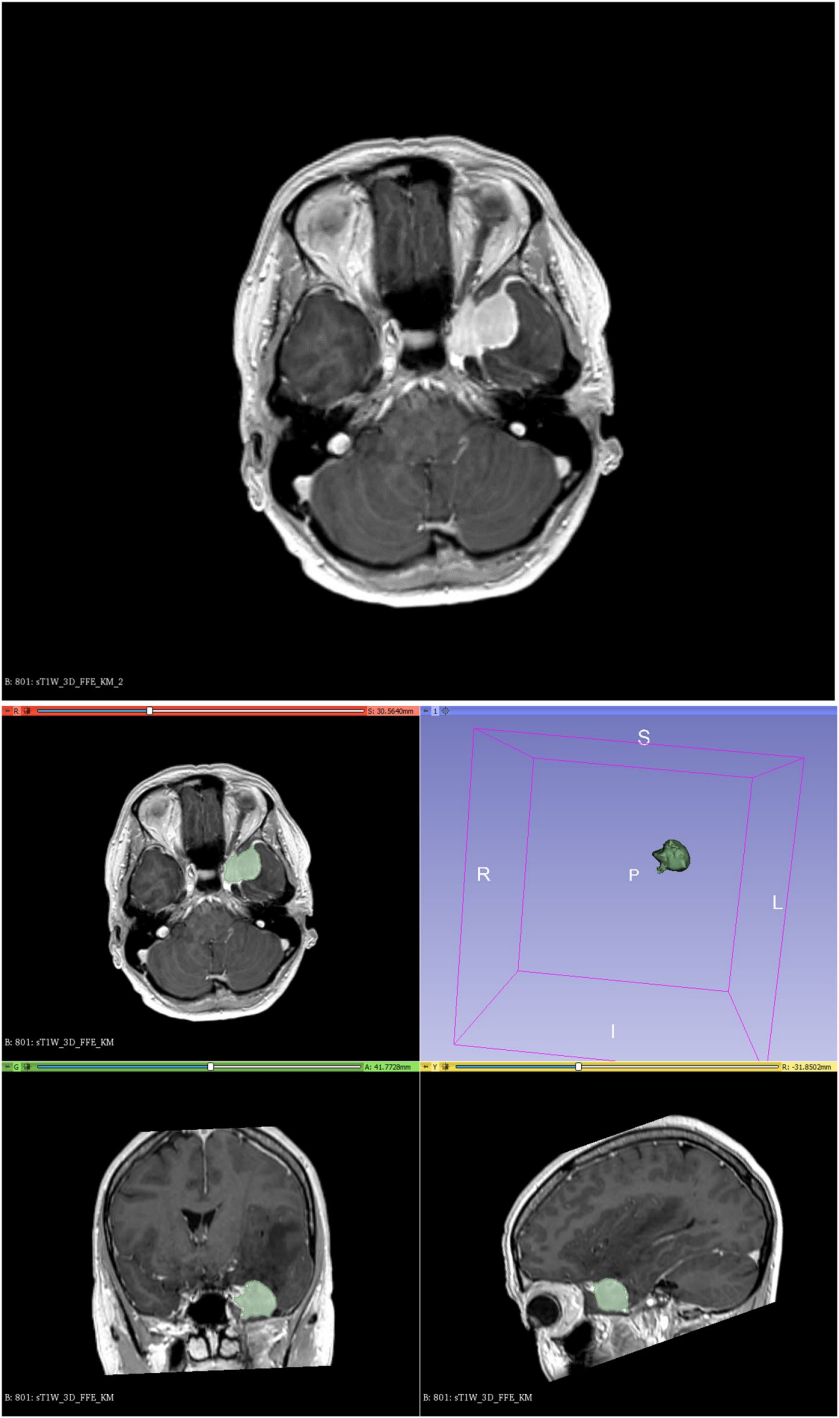
Meningioma of the skull base (above); semi-automatic segmentation with 3D Slicer (below).

Segmentation of the contrast enhancing parts of the tumor was semi-automatically performed using the 3D Slicer open-source software platform (version 4.10, www.slicer.org) and utilizing the Segmentation Wizard plugin. As an example, Fig. 1 shows a meningioma of the skull base. The figure includes the semi-automatic segmentation with 3D Slicer. Two readers with 5 and 9 years of experience in neuroradiology drew the segmentation. Consensus was achieved in cases of differing extent of segmentation. We performed a standardized preprocessing step on all images: first spatial resampling to 2 × 2 × 2 voxels, then a bin width of 64 was set. For the computation of the radiomics features we used the open source PyRadiomics package available as an implementable plugin into the 3D Slicer platform. The segmentation tool allows the segmentation as a volume of interest (VOI). The open source software allows for direct calculation of 3D features. No combination or averaging across slices is necessary. We extracted a total of 107 radiomic features by hand-delineated regions of interest (ROI) from the MRI images of each patient. These 107 radiomic features belong to seven different features classes: 18 first order statistics, 14 shape-based features, 24 Gy level co-occurrence matrix, 16 Gy level run length matrix, 16 Gy level size zone matrix, 5 neighbouring gray tone difference matrix and 14 Gy level dependence matrix. In addition, our database contained further factors, such as gender and age, the location of the meningioma, shape and subtype of the tumor, the distinction between a first diagnosis of the tumor and a relapse and the **K**arnofsky **P**erformance scale **I**ndex (KPI). All categorical variables were used in binary form. For example, if a meningioma was located in the falx, the value of the feature "Tumor.location.falx" was equal to 1, otherwise equal to 0. All features were z-score transformed and then subjected to a 95% correlation filter to account for redundancy between the features. Highly correlated features contain a significant amount of identical information. Therefore, considering highly correlated variables simultaneously in a model does not add significant value compared to considering only one of the features from this group. We analyzed the discriminatory power (p-value) for each feature.

### Statistical analysis

Statistical analysis was performed using R software (version 3.5.3). As mentioned above, contrast-enhanced T1-weighted images before meningioma resection were available for 138 patients. These 138 patients were allocated to training data and independent test data at random. Specifically, a stratified 4:1 ratio (training data: 111 patients, test data: 27 patients) was used with a balanced distribution of GTR/STR and gender (female/male) between the two samples (Table 1). The stratified division of the data into the two groups was performed using the “splitstackshape” package in R. Importantly, patients were split into training and test data 100 times for each model. Thus, in total, we used 100 different training and 100 different test samples. The training data sets were used to construct different machine learning models and to optimize the tuning parameters included in these models. The performance of the models was then determined with the corresponding test data sets (i.e. using unknown/independent data).

**Table 1:** Clinical and demographic characteristics.

	Training	Independent	Total
	Data	Test data	Data
Number of patients	111	27	138
**Resection status (in %)**			
GTR	77.48	77.78	77.54
STR	22.52	22.22	22.46
Mean age (in years)	58.80	59.12	58.86
**Gender (in %)**			
Male	27.93	25.93	27.54
Female	72.07	74.07	72.46
**Tumor location (in %)**			
Convexity	33.32	33.41	33.33
Falx	12.35	12.19	12.32
Skull base	46.57	45.59	46.38
Posterior fossa	7.77	8.81	7.97
**Tumor shape (in %)**			
Irregular	37.87	36.89	37.68
Regular	62.13	63.11	62.32

### Feature preselection and model construction

**Figure 2:**
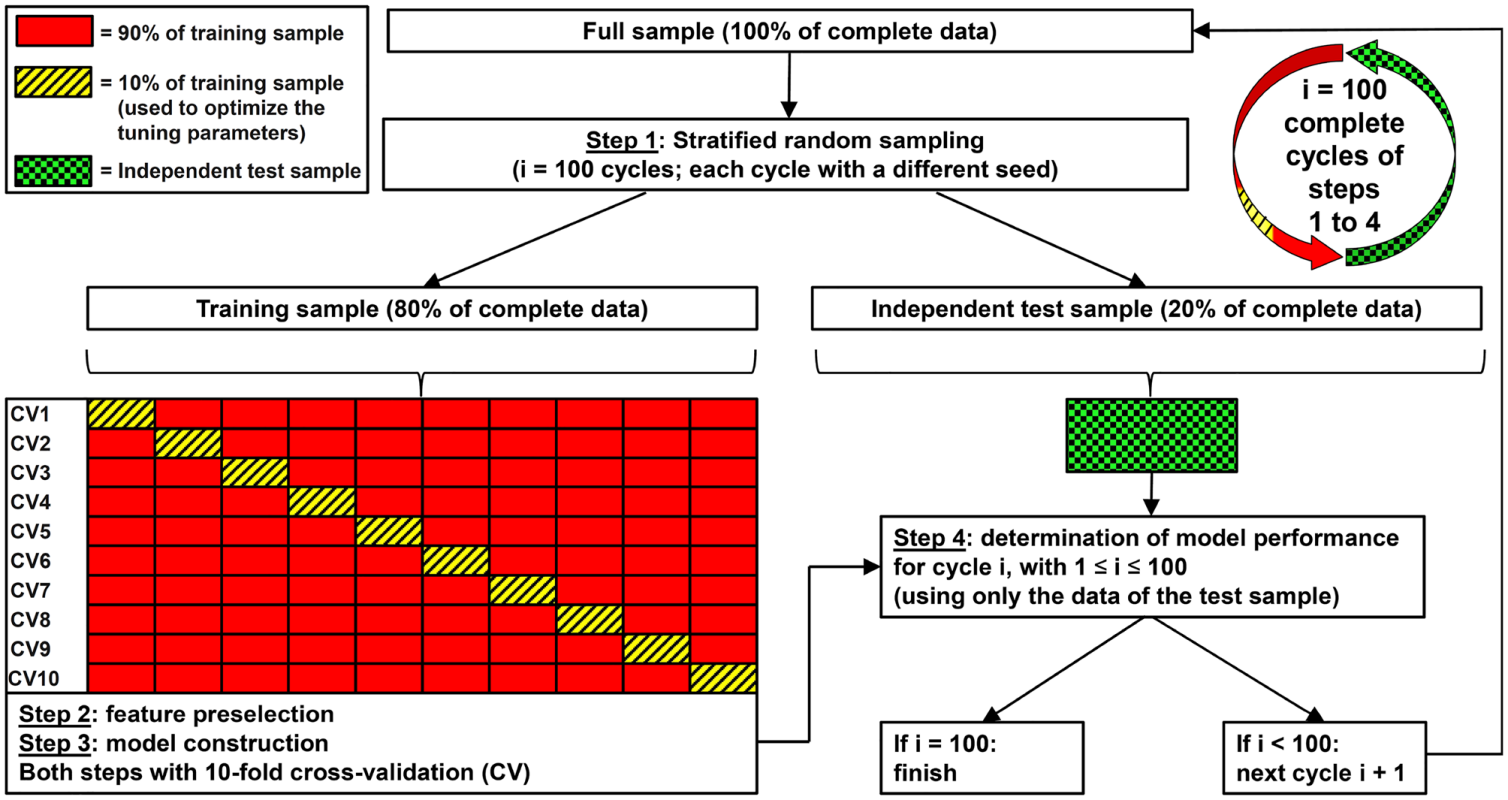
Development and test of a model with 100 repetitions (100 cycles), fixed number of features und a fixed machine learning algorithm used for feature preselection and for the subsequent model estimation.

We used a four-step approach to construct and test our models (see Fig. 2). In the first step, the full dataset was split into a training and a test sample. In the second step, the feature preselection of the most important features was performed. Therein, the “varImp” function in R was used to identify these most important (most discriminant) features. This function determines the additional performance of each feature included in a model, i.e. the performance of a model is calculated with and without this feature. The difference in the performance of these two models then determines the performance gain resulting from this feature. The feature that causes the highest performance loss when removed from the model has the highest importance. In the third step, the models containing the features identified in the second step were estimated, using the same machine learning method. Finally, in the fourth step, the models were tested using the corresponding unknown/ independent test sample. This four-step approach was completely repeated 100 times for each model using the 100 different training and test data sets as explained above. The 100 repetitions were performed to exclude possible overfitting of the models and random effects related to data partitioning.

Feature preselection (step 2) and subsequent model construction (step 3) was performed with eight different machine learning algorithms using only the training data:

1. Logistic regression
2. Lasso regression
3. Ridge regression
4. Random forest
5. Bagged trees
6. **G**radient **B**oosting **M**achine (GBM)
7. Naive Bayes
8. **L**inear **D**iscriminant **A**nalysis (LDA)

We created our models with an increasing number of the most important features identified in each second step. Initially, each model contained only the most important feature, followed by a model with the two most important features, followed by a model with the three most important features, and so on. The model with the highest mean performance with respect to the independent test data was used as the final model. This step-by-step approach determined the final number of features included in the model. The approach described here was performed independently for each of the eight machine learning algorithms listed above. The aim of this approach with an increasing number of features was to find and select the most highly discriminating multivariate feature combination with as few features as possible. The model optimization was performed for all models by maximizing of the **A**rea **U**nder the **C**urve (AUC) of the **R**eceiver **O**perator **C**haracteristic (ROC). The predictive power of each model was analysed using AUC, accuracy, sensitivity, specificity and Cohen’s **Kappa** (kappa = (observed accuracy – expected accuracy) / (1- expected accuracy)). Given the large imbalance in our class distribution of more than 3:1 (GTR vs. STR cases), Cohen's Kappa provides a more objective description of model performance than accuracy. Higher, i.e. better values (closer to the + 1 value) for Cohen's Kappa are much more difficult to achieve if the class distribution is unbalanced. The opposite is true for accuracy. In addition to the aforementioned performance measures, the performance of the final model was analysed in relation to the location of the tumor in the skull and the postoperative tumor volume.

For both, the eight different methods of feature preselection and subsequent model constructions only the training data were used. The tuning parameters of our models were determined using a tenfold cross validation (i.e. we divided the training data 10 times into groups with 90% and 10% of the training data, respectively). This technique ensures that the subgroups of the training data do not overlap. It is a methodology often used to obtain robust results with small datasets. We thus performed a double cross-validation overall, so to speak. On a first level for the division of the data into training and test data and the subsequent determination of the performance with the independent test data and on a second level for the determination of the hyperparameters using only the training data.

In summary, each of our models with a fixed number of features and a fixed machine learning algorithm was fully constructed, estimated and tested 100 times. For this purpose, first the stratified division of the full dataset into training data (80% of data) and test data (20% of data) described above was repeated *i* = 100 times (step 1 in Fig. 2) using different seeds for the data partitioning. This means that we used 100 different training samples and 100 different test samples with unknown data to develop and test each model. Variable preselection was then performed with each of these training samples (step 2), then each of the 100 models was estimated using the respective training sample number *i* (step 3), and finally each final model number *i* was tested with the respective independent test sample number *i* (step 4). The complete process for developing and testing a single model with a fixed number of features and a fixed machine learning algorithm is shown in Fig. 2. We performed this complex approach with 100 replicates, firstly to eliminate overfitting as far as possible and secondly to be able to determine how sensitively the performance of the models, as well as the selection of the features included in the models, depend on the samples used.

## Results

### Determination of the most important features

First, we analysed the importance of the different features depending on the algorithm used for the feature preselection. The five most important features for each of the eight different algorithms are listed in Table 2. The features with the ranks of importance 6 to 10 can be found in the supplemental (Supplementary Table 1). It is interesting to note that the eight different methods used to determine the most important (most discriminant) features yield almost the same features. Most of the first features are non-radiomic features.

**Table 2:** Most important five features for each of the eight feature preselection methods.

Feature pre-selection method	Rank of feature importance				
	**1**	**2**	**3**	**4**	**5**
Stepwise logistic	Tumor shape: irregular or regular	Tumor location: convexity	Tumor location: falx	orig.shape.Elongation	fd_vs_re: first diagnose or relapse
Lasso	Tumor shape: irregular or regular	Tumor location: skull base	orig.shape.Elongation	fd_vs_re: first diagnose or relapse	Tumor location: posterior fossa
Ridge	Tumor shape: irregular or regular	Tumor location: convexity	Tumor location: falx	Tumor location: skull base	orig,shape,Elongation
GBM	Tumor shape: irregular or regular	Tumor location: skull base	Tumor location: convexity	orig,glszm.SizeZone NonUniformity	orig,shape,Elongation
Random forest	Tumor location: convexity	Tumor location: skull base	orig.glszm.SizeZone NonUniformity	Shape: irregular or regular	fd_vs_re: first diagnose or relapse
Bagged trees	Tumor location: skull base	Tumor location: convexity	orig.shape.Sphericity	Shape: irregular or regular	orig.shape.Elongation
LDA	Tumor shape: irregular or regular	Tumor location: convexity	Tumor location: skull base	KPI	orig.glszm.Small AreaEmphasis
Naive Bayes	Tumor shape: irregular or regular	Tumor location: convexity	Tumor location: skull base	KPI	orig.glszm.Small AreaEmphasis

**Figure 3:**
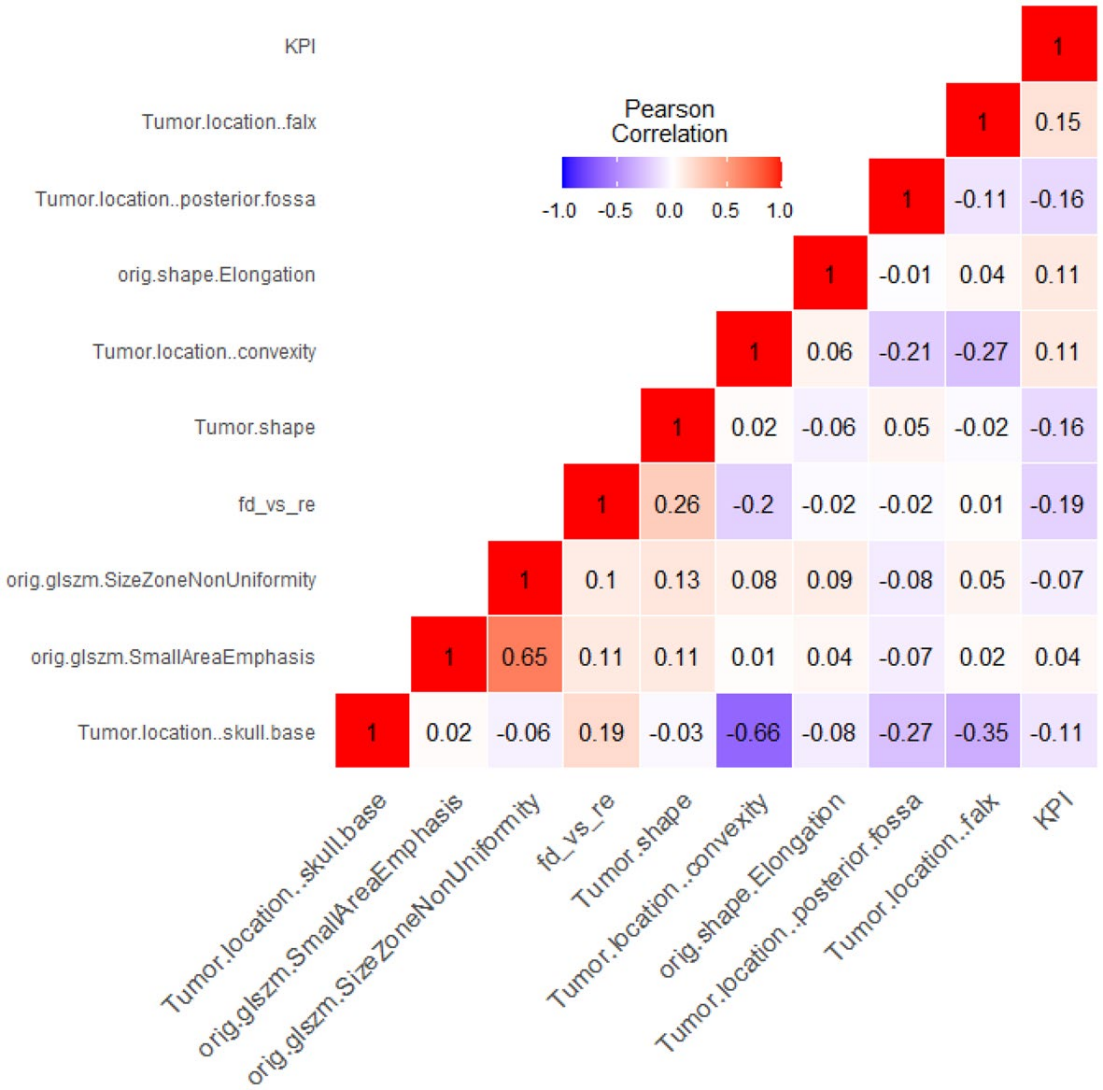
Pearson correlation matrix for the 10 most frequently selected features.

Almost all of the features listed in Table 2 are assumed to be statistically significant discriminant. Using chi-square test (Fisher’s exact test) for binary and categorical features we received p-values < 0.001 for the tumor locations skull base and convexity, the tumor shape (irregular or regular) and the feature “fd_vs_re” (first diagnose or relapse). The p-values for the location = falx and the **K**arnofsky **P**erformance scale **I**ndex (KPI) were 0.039 and 0.046. Using Wilcoxon test (Mann*–*Whitney*-*U*-*Test), the *p* values for the non-normally distributed continuous features “orig.glszm.SmallAreaEmphasis”, “orig.shape.Elongation” and “orig.glszm.SizeZoneNonUniformity” were 0.048, 0.059 and 0.128 respectively. The two additional features "age" and "gender" in Table 1, on the other hand, have no univariate statistically relevant (*p* values > 0.05) discriminatory power.The shape of the tumour (irregular or regular) and its location are particularly important. In our database, we distinguish the following four locations of meningiomas in the brain: convexity, falx, skull base (see Fig. 1) and posterior fossa. Other multivariate important features are the distinction between a first diagnosis of the tumor and a relapse (variable “fd_vs_re”), the KPI and the radiomic features “orig.shape.Elongation”, “orig.glszm.SmallAreaEmphasis” and “orig.glszm.SizeZoneNonUniformity. “The correlation matrix for these 10 most frequently selected features is shown in Fig. 3. Most of the correlation coefficients are small. This means that most of the features are only slightly dependent on each other. The variables “orig.shape.Elongation” and “orig.glszm.SizeZoneNonUniformity “show the highest positive correlation ($\varrho$ = 0.65) and the locations convexity and skull base the highest negative correlation ($\varrho$ = – 0.66). The reason for the comparatively high negative correlation coefficient with respect to the locations convexity and skull base is simply that the individual tumor locations are mutually exclusive, and tumor locations convexity and skull base occur most frequently in our data. In general, variables with high correlation coefficients should not be used together in a multivariate model.

### Performance results of the different models

**Figure 4:**
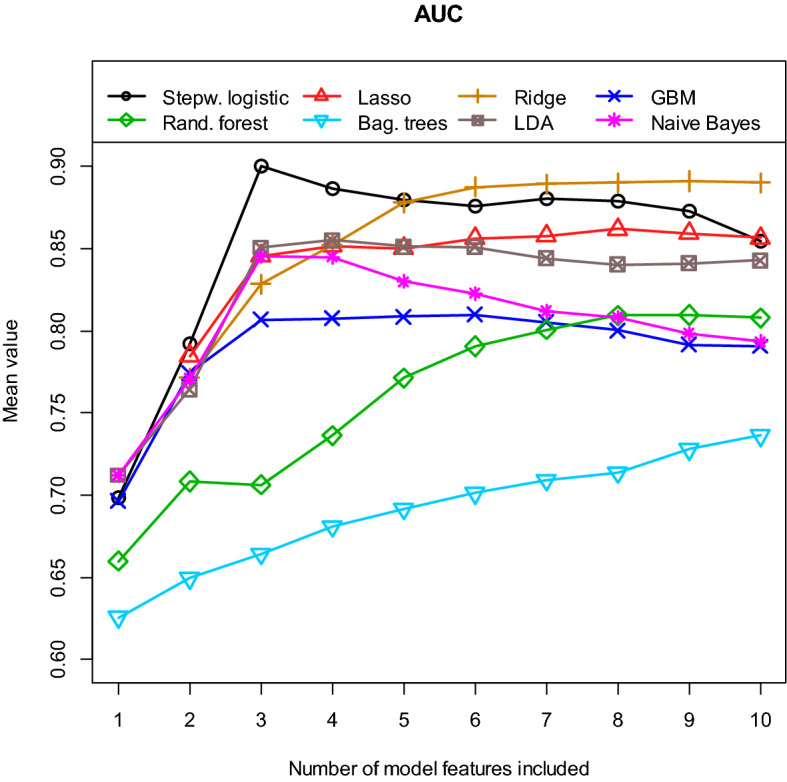
**A**rea **U**nder the **C**urve **(AUC)** for the test samples, calculated as means of 100 repetitions (100 cycles).

**Figure 5:**
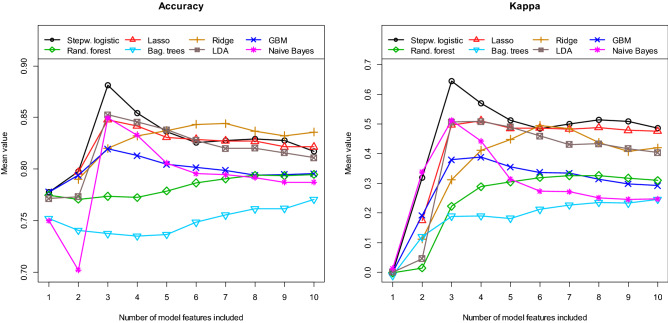
**Accuracy** and **Kappa** for the test samples, calculated as means of 100 repetitions (100 cycles).

**Figure 6:**
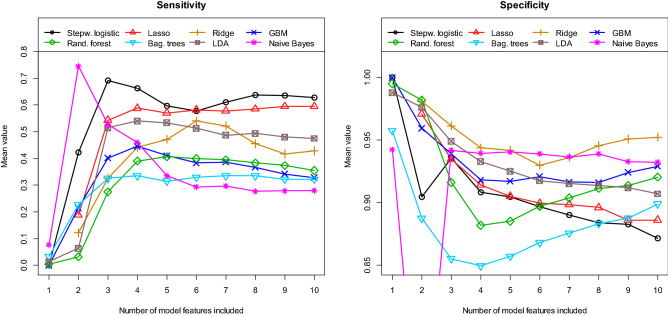
**Sensitivity** and **Specificity** for the test samples, calculated as means of 100 repetitions (100 cycles).

Next, we determined the performance of our different multivariate models. Figure 4 shows the obtained AUC values for the eight different machine learning algorithms as a function of the number of model features included. Table 2 and Supplementary Table1 indicate which features are most frequently included in each model. A model with n features contains the first/most important n features in each case. Since each model was re-estimated 100 times, the features used in the individual model (per cycle) may differ from the tables. All values in Fig. 4 are calculated as means of the 100 cycles/repetitions using the independent test samples. The best value of mean AUC = 0.900 [0.786, 0.976] is obtained with the stepwise logistic regression model, which contains only three features. The values in the brackets indicate the 95% confidence interval (CI). For almost all model approaches shown here, the use of more than about 6 features does not lead to a further significant increase in the discriminatory power. In Fig. 5 the received values for the mean accuracy and for the mean kappa are shown. Again, the best performance is obtained with the logistic model containing only three features. For this model, the mean accuracy is 0.881 und the mean kappa is 0.644. Finally, Fig. 6 summarises the results for the mean sensitivity (this means correct prediction of the STR cases) and the mean specificity (this means correct prediction of the GTR cases). In terms of the mean sensitivity, the logistic model with three features is only the second best of our models. The Naive Bayes approach with only two features leads here to an even slightly higher value with a mean sensitivity of 0.745. High mean specificities are obtained with almost all models. Overall, we obtained our worst results in terms of AUC, accuracy, kappa, sensitivity, and specificity with the bagged trees algorithm and the random forest model. It is interesting to note that the radiomic features have only a minor influence on the logistic model with three variables. For the other algorithms, however, their significance is higher. This is especially true for the Ridge regression, random forest and bagged trees algorithm.

### Classification results of the final model: logistic model with three features

The logistic model, which contains only three variables, performed well on all our performance measures. The classification results with the training data and the test data, calculated as the means of the 100 repetitions, are summarized together with their 95% confidence intervals in Table 3. It is interesting to note that our technique with 100 repetitions produces very robust results for the logistic model used here. For the training data and the test data, the mean results are extremely similar. As follows from Fig. 4, performance with the independent test data is worse with both more than three and less than three variables. It is therefore very likely that there is no overfitting in the model with three variables.

**Table 3:** Classification results of the logistic regression model with three features for training data and independent test data, calculated as means of 100 repetitions (100 cycles). Values in brackets: 95% confidence interval.

	Training data	Test data
AUC	0.901 [0.879, 0.926]	0.900 [0.786, 0.976]
Accuracy	0.875 [0.856, 0.896]	0.881 [0.778, 0.963]
Kappa	0.629 [0.562, 0.692]	0.644 [0.348, 0.899]
Sensitivity	0.675 [0.600, 0.760]	0.692 [0.333, 1.000]
Specificity	0.933 [0.919, 0.953]	0.936 [0.857, 1.000]

Regarding the patients in our database, meningiomas with location convexity and falx could be completely resected (i.e. GRT cases) without exceptions. However, most meningiomas with location skull base and posterior fossa could not be completely resected (i.e. STR cases) if they had an irregular shape and could also be completely resected (i.e. GTR cases) if they had a regular shape. These usual clinical outcomes, as well as the prediction error rates of our three-factor model using the independent test samples, are summarized in Table 4. Almost all prediction errors of our relatively simple model occur for meningiomas localised in the skull base or posterior fossa. Here, the model predicts usually incorrectly if a meningioma with irregular shape could be completely resected as well as if a meningioma with regular shape could not be completely resected. All other cases are almost always correctly predicted.

**Table 4:** Usual clinical outcomes and prediction error rates (using the test samples) for the stepwise logistic regression model with three features.

Tumor location	Usual clinical outcome		Prediction error rate	
	Shape: irregular	Shape: regular	Shape: irregular	Shape: regular
Convexity	GTR	GTR	0.00%	0.00%
Falx	GTR	GTR	2.48%	0.00%
Skull base	STR	GTR	24.25%	21.55%
Posterior fossa	STR	GTR	22.32%	11.90%

**Figure 7:**
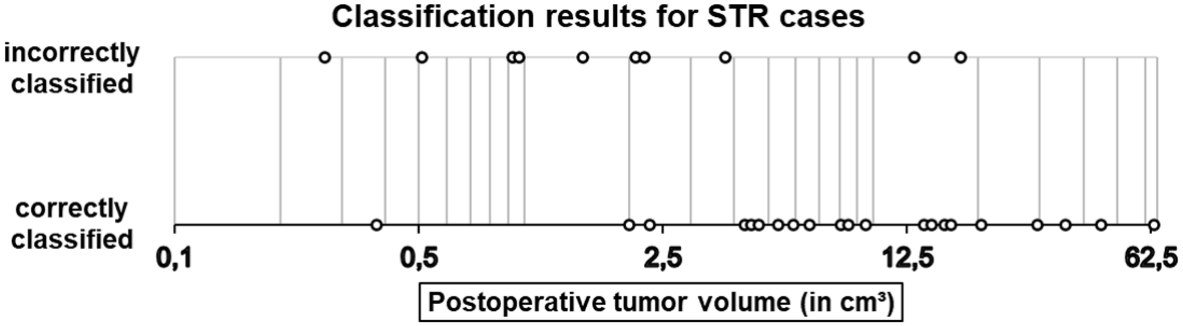
Classification results of the logistic regression model with three features for the STR cases, calculated with the test samples.

We analysed these misclassified cases in even more detail. Regarding the GTR cases, the error rate using the independent test data is only 6.43%, which means that the model has a very high specificity of 0.9357. However, the prediction error for the STR cases shows a strong dependence on the postoperative tumor volume (POTV). With respect to the cases with the smallest eleven POTVs, a total of eight cases were misclassified by our model. These eight patients had only small POTVs: 4 cases with a POTV < 1.0 cm^3^, another 3 cases with a POTV < 2.25cm^3^ and one more case with a POTV = 3.78 cm^3^. All other 21 STR cases were correctly classified, except for 2 additional cases (POTVs = 13.1 cm^3^ and 17.9 cm^3^). Figure 7 shows the classification results as a function of postoperative tumor volume for the STR cases. The POTV is shown logarithmically scaled according to the vertical lines. The small dots in the figure indicate whether the respective case was correctly or incorrectly classified. It is obvious that the prediction error for the STR cases depends strongly on the POTV. The sensitivity for the correct prediction of STR cases is 0.6917. However, the sensitivity in predicting cases with a POTV > 4 cm^3^ is as high as 0.8980. Thus, our model is able to correctly predict almost all cases except those with a very small POTV.

## Discussion

Our results show that it is possible to predict possible complete tumor resections (GTR) or subtotal tumor resections (STR) before treatment with good discriminatory power using machine learning algorithms. Using a three-factor logistic model, we achieved a very high and stable performance in discriminating GTR and STR cases with independent test data (AUC = 0.900, accuracy = 0.881, kappa = 0.644, sensitivity = 0.692 and specificity = 0.936). In multivariate logistic models, the features included in a model can be interpreted easily in terms of their direction of effect. As explained, every model was created 100 times, estimated with 100 different training samples and subsequently tested with 100 sets of independent test samples. Each of our 100 models with three features created with stepwise logistic regression contained the shape of the tumor (feature “Tumor.shape”) and different possible locations of the meningiomas. In most models, the locations convexity and falx were included. Sometimes, locations skull base and posterior fossa were included instead. Our model shows that even with these few features, a fairly accurate statement can be made about whether a meningioma can be completely resected or not. However, cases with very small postoperative tumor volumes (< 4 cm^3^) are difficult to predict.

The tumors in our database that could only be resected subtotally were located at the skull base and in the posterior fossa. Wang et al. found that the extent of the surgical resection of a meningioma located at the skull base significantly influenced the prognosis. GTR of meningioma improved progression-free survival compared to STR^9^. As early as 1957, Simpson described the extent of meningioma resections in a grading system. He showed that the extent of surgical resection and tumor recurrence are correlated^10^. According to Voß et al., increasing Simpson grade and subtotal resection are still generally correlated with tumor recurrence^11^. Gallagher et al. also found, that Simpson grade remains a predictive factor for recurrence/progression free survival (RPFS). However, the meningioma location no longer appears to be a significant predictor of RPFS. They hypothesize that this may be due to the increased use of adjuvant therapies for skull base meningiomas, as well as advances in technology and surgical techniques^12^. Lemée et al. analyzed risk factors for incomplete resections, using a cohort of 1469 patients. In line with our results, they found that a location of the tumor at the skull base was one of the most important risk factors. In addition, they identified two further important factors which were not included in our database: symptoms at presentation (seizure, intracranial hypertension and/or a neurological deficit) and associated bone invasion^13^. In our study, we found that in addition to meningiomas located at the skull base, tumors in the posterior fossa could comparatively often not be completely resected. Specifically, in our final dataset 40.6% of skull base meningiomas and 45.5% of posterior fossa meningiomas were STR cases. This finding is also in line with several other studies^14,15^. Corniola et al. identified factors and provided a classification tree to predict the EOR in posterior fossa meningiomas, based upon preoperative demographic, clinical, and radiological variables^16^.

These studies underline the well-known fact that the degree of resection is an important parameter for early further therapy planning. In our study, we presented a methodology to develop stable models with high discriminatory power using machine learning methods to predict and discriminate between gross total and subtotal resections. We used an algorithm with 100 cycles/repetitions and a variable number of features to avoid both underfitting and overfitting. As the comparison of the performance values achieved with the training data and the test data shows (Table 3), this methodology achieved extremely stable results. Specifically, our method resulted in a model with high discriminative power using only three features.

Our study has some limitations. First of all, the final cohort was relatively small including only 138 patients. This is especially true for the STR cases. In order to be able to classify these cases even more precisely, more corresponding STR cases are needed to train the model. In addition, further information concerning the patients would be beneficial, e.g. whether there is bone invasion of the tumor. Finally, the retrospective character should be mentioned. Regardless of these limitations, this study demonstrates how machine learning algorithms can be used to predict clinical resection outcomes, potentially accelerating further treatment planning.

## Conclusion

Our results show that with machine learning algorithms and comparatively few explanatory factors, it is possible to make accurate predictions about whether a meningioma can be completely resected by surgery or not. Complete resections as well as resections with larger postoperative tumor volumes can be predicted with very high accuracy. However, cases with very small postoperative tumor volumes are comparatively difficult to predict correctly.

## Supplementary Information

Supplementary Information.

## Data Availability

The datasets used and/or analysed during the current study available from the corresponding author on reasonable request.

## References

[1] Marosi C (2008). Meningioma. Crit. Rev. Oncol. Hematol..

[2] Alshibany AM, Al-Husaini HH (2021). Late recurrence of metastatic meningioma in the lung in a patient with endometrial cancer: A case report. Am. J. Case Rep..

[3] Apra C, Peyre M, Kalamarides M (2018). Current treatment options for meningioma. Expert Rev. Neurother..

[4] Buerki RA (2018). An overview of meningiomas. Future Oncol. Lond. Engl..

[5] Hunter JB (2018). Tumor progression following petroclival meningioma subtotal resection: A volumetric study. Oper. Neurosurg. Hagerstown Md.

[6] Heo J (2019). Machine learning-based model for prediction of outcomes in acute stroke. Stroke.

[7] Lee YW, Choi JW, Shin E-H (2021). Machine learning model for predicting malaria using clinical information. Comput. Biol. Med..

[8] Park YW (2019). Radiomics and machine learning may accurately predict the grade and histological subtype in meningiomas using conventional and diffusion tensor imaging. Eur. Radiol..

[9] Wang Y-C (2015). Skull base atypical meningioma: long term surgical outcome and prognostic factors. Clin. Neurol. Neurosurg..

[10] Simpson D (1957). The recurrence of intracranial meningiomas after surgical treatment. J. Neurol. Neurosurg. Psychiatry.

[11] Voß KM (2017). The Simpson grading in meningioma surgery: does the tumor location influence the prognostic value?. J. Neurooncol..

[12] Gallagher MJ, Jenkinson MD, Brodbelt AR, Mills SJ, Chavredakis E (2016). WHO grade 1 meningioma recurrence: Are location and Simpson grade still relevant?. Clin. Neurol. Neurosurg..

[13] Lemée J-M (2019). Extent of resection in meningioma: Predictive factors and clinical implications. Sci. Rep..

[14] Roberti F, Sekhar LN, Kalavakonda C, Wright DC (2001). Posterior fossa meningiomas: Surgical experience in 161 cases. Surg. Neurol..

[15] Lobato RD (2004). Meningiomas of the basal posterior fossa. Surgical experience in 80 cases. Neurocir. Astur. Spain.

[16] Corniola MV (2019). Posterior fossa meningiomas: Perioperative predictors of extent of resection, overall survival and progression-free survival. Acta Neurochir. (Wien).

