# Supplementary material for: Assessing preoperative risk of STR in skull meningiomas using MR radiomics and machine learning
Source: Sci Rep. 2022 Aug 18;12:14043. doi: 10.1038/s41598-022-18458-4 (PMC9388514; doi:10.1038/s41598-022-18458-4)
Supplement: Supplementary file 1 — Supplementary Information. [file 41598_2022_18458_MOESM1_ESM.docx]

**Supplemental**

| **Feature pre-** | **Rank of feature importance** | | | | |
| --- | --- | --- | --- | --- | --- |
| **selection method** | **6** | **7** | **8** | **9** | **10** |
| **Stepwise logistic** | gender:  male or female | age:  in years | orig,glcm,Cluster  Prominence | orig,glszm,LargeAreaHighGrayLevelEmph | orig,shape,Max,  2D.DiametColumn |
| **Lasso** | gender:  male or female | Tumor location:  convexity | age:  in years | orig,glszm,LargeAreaHighGrayLevelEmph | orig,fst,ord,Energy |
| **Ridge** | fd_vs_re: first  diagnose or relapse | gender:  male or female | Tumor location:  posterior fossa | orig,fst,ord,Energy | age:  in years |
| **GBM** | orig,glrlm,RunLengthNonUniformity | orig,glszm,SmallAreaLowGrayLevEmphasis | fd_vs_re: first  diagnose or relapse | orig,glcm,Idmn | pre. op. tumor volume |
| **Random forest** | orig,shape,Elongation | orig,glszm,SmallAreaEmphasis | orig,glrlm,RunLengthNonUniformity | orig,shape,Major  AxisLength | Tumor location:  falx |
| **Bagged trees** | orig,shape,Max,  2D.DiametColumn | orig,shape,Voxel  Volume | orig,glszm,Size  ZoneNonUniformity | age:  in years | orig,shape,Surface  VolumeRatio |
| **LDA** | KPI:  <= 80 or > 80 | fd_vs_re: first  diagnose or relapse | orig,shape,Major  AxisLength | orig,glszm,ZoneEntropy | orig,glszm,Size  ZoneNonUniformity |
| **Naive Bayes** | KPI:  <= 80 or > 80 | fd_vs_re: first  diagnose or relapse | orig,shape,Major  AxisLength | orig,glszm,ZoneEntropy | orig,glszm,Size  ZoneNonUniformity |

**Table 1:** Features with ranks of importance 6 to 10 for each of the eight feature preselection methods
